# Modulating root system architecture: cross-talk between auxin and phytohormones

**DOI:** 10.3389/fpls.2024.1343928

**Published:** 2024-02-08

**Authors:** Mehmood Jan, Sajid Muhammad, Weicai Jin, Wenhao Zhong, Shaolong Zhang, Yanjie Lin, Yueni Zhou, Jinlong Liu, Haifeng Liu, Raheel Munir, Qiang Yue, Muhammad Afzal, Guoping Wang

**Affiliations:** ^1^ College of Horticulture, South China Agricultural University, Guangzhou, Guangdong, China; ^2^ Guangdong Provincial Key Laboratory of Utilization and Conservation of Food and Medicinal Resources in Northern Region, Shaoguan University, Shaoguan, China; ^3^ College of Agriculture, South China Agricultural University, Guangzhou, Guangdong, China; ^4^ Department of Agronomy, College of Agriculture and Biotechnology, Zhejiang University, Hangzhou, China; ^5^ Heyuan Division of Guangdong Laboratory for Lingnan Modern Agricultural Science and Technology, Heyuan, Guangdong, China

**Keywords:** auxin crosstalk, auxin-CK, auxin-SL interaction, phytohormones, root architecture, root development

## Abstract

Root architecture is an important agronomic trait that plays an essential role in water uptake, soil compactions, nutrient recycling, plant–microbe interactions, and hormone-mediated signaling pathways. Recently, significant advancements have been made in understanding how the complex interactions of phytohormones regulate the dynamic organization of root architecture in crops. Moreover, phytohormones, particularly auxin, act as internal regulators of root development in soil, starting from the early organogenesis to the formation of root hair (RH) through diverse signaling mechanisms. However, a considerable gap remains in understanding the hormonal cross-talk during various developmental stages of roots. This review examines the dynamic aspects of phytohormone signaling, cross-talk mechanisms, and the activation of transcription factors (TFs) throughout various developmental stages of the root life cycle. Understanding these developmental processes, together with hormonal signaling and molecular engineering in crops, can improve our knowledge of root development under various environmental conditions.

## Introduction

Plant architecture is considered the most important trait that affects stress resistance and yield (Guo et al., 2020a). Although the above-ground parts (green parts) of plants participate vigorously in photosynthesis and transpiration (Sharma et al., 2021), reduction in plant height, leaf width, and length, branching pattern, and even the angles of the leaves have a significant effect on light capturing, photosynthesis, and, finally, the plant’s development and growth (Berry and Argueso, 2022). Moreover, the changes in these traits can directly affect the aptitude of the crops to grow in high-density planting (HDP) systems and their mechanical harvesting at the end (Huot et al., 2014; Berry and Argueso, 2022). For example, in maize, the sparsely and upright-branched phenotype is better for HDP (Tian et al., 2019); however, in rice, the semi-dwarf phenotype overcomes lodging (Lo et al., 2017; Wang et al., 2017).

Similarly, the hidden parts of the plants (roots) control the wide-ranging processes that are essential for the regular development and growth of plants, such as water acquisition, nutrient uptake from soil and transfer to other parts, and soil anchorage, and also for mechanical support (Li et al., 2018). Additionally, roots also form a site for the symbiotic relationship of plant–microbe interaction, such as nitrogen fixation (Afzal et al., 2022). Root architecture (RA) optimization mainly depends upon the plant species, environmental conditions, and the demand from the green parts of the plants (Hodge et al., 2009). Therefore, modifications in RA, such as reduced length of primary roots (PRs), changes in structure and numbers of the lateral roots (LRs), and root hairs (RHs) preclude crops from regularly obtaining water and the uptake of vital nutrients from the soil for normal development. In the current era, the main challenge for crop breeders in modern agricultural practices is producing an ideal plant with improved plant architecture suitable for more yield under various environmental conditions.

Owing to the sessile condition of plants, understanding the RA would be helpful for improving yield and optimization for agricultural land. The usage of newly developed technologies, such as three-dimensional (3-D) root imaging, advances in digital photography, fluorescence-based imaging systems, and X-ray tomography, and using transparent soil have assisted scientists in understanding the complex system of RA in different plant species (Tötzke et al., 2019). Similarly, with advancements in the new techniques of modern molecular biology, the identification of the important gene and signaling pathways in plant species has become a reality (Šimášková et al., 2015).

In addition, plant hormones regulate numerous metabolic pathways to facilitate plant adoption in harsh environments. In different plant species, many mutants of hormonal biosynthesis and signaling have been identified and used to investigate the importance of hormones in plant architecture under different environmental conditions (Iqbal, 2014; EL Sabagh et al., 2022). For instance, gibberellin is documented to be shown as a regulator effect on root development. The study on gibberellin-deficient *Arabidopsis* mutants showed reduced root elongation, which was connected with lower production of gibberellin in roots (Willige et al., 2007). Correspondingly, numerous studies demonstrated the cross-talk between phytohormones such as auxin and gibberellic acid (GA) (Niu et al., 2013). Auxins have been noted to modulate the expression of GA synthesis-related genes in several plant species (Frigerio et al., 2006), while GA is involved in increasing the polar-auxin transport in plants (Björklund et al., 2007). Similarly, auxin signaling was noted in the regulation of the RGA (repressor of *ga1-3*), thus indorsing GA signaling, and root development (Fu and Harberd, 2003). Moreover, the exogenous application of GA was reported as an RH promotor.

Plant RA is influenced by a complex interaction of regulatory networks that involve phytohormones, transcription factors (TFs), and miRNAs (Tang and Chu, 2017; Li et al., 2018). In addition, phytohormone signaling pathways and their cross-talk govern the developmental programs of whether to begin, continue, or maybe arrest. Therefore, this review aims to elucidate the potential role of phytohormone-regulated TFs, which results in root development and their possible interaction for ideal RA. Gaining insights into these developmental processes, together with engineering their expression and activity of related factors, can enhance our understanding of creating smart crops under various environmental conditions.

## Phytohormones as significant regulators in root architecture

**Figure 1:**
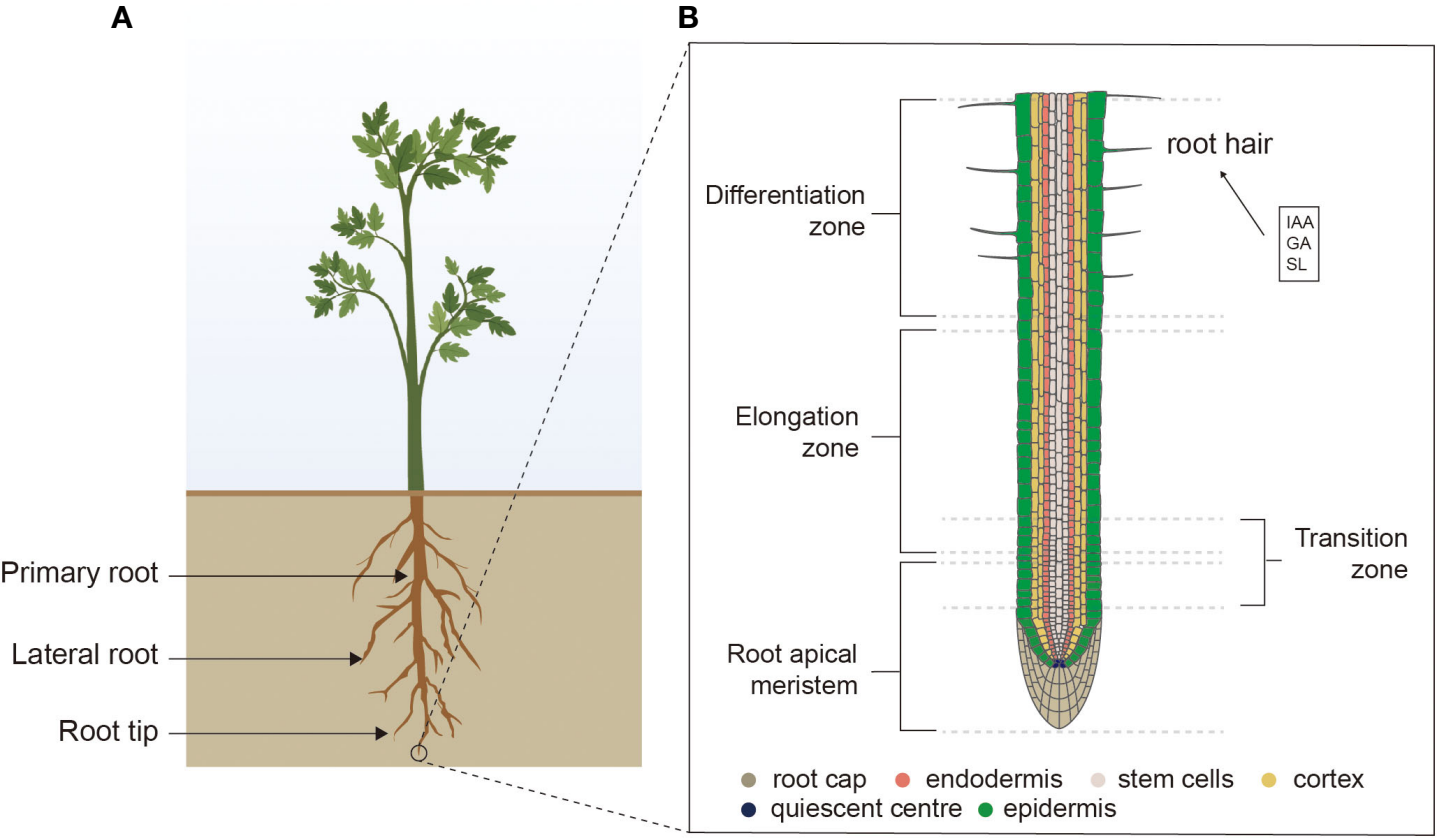
The tap root system. **(A)** RSA of dicots representing different parts of roots. **(B)** Root tip showing root cap, endodermis, stem cells, cortex, quiescent center, epidermis, root apical meristem, elongation zone, transition zone, differentiation zone, and RHs. RSA, root system architecture; RHs, root hairs.

**Figure 2:**
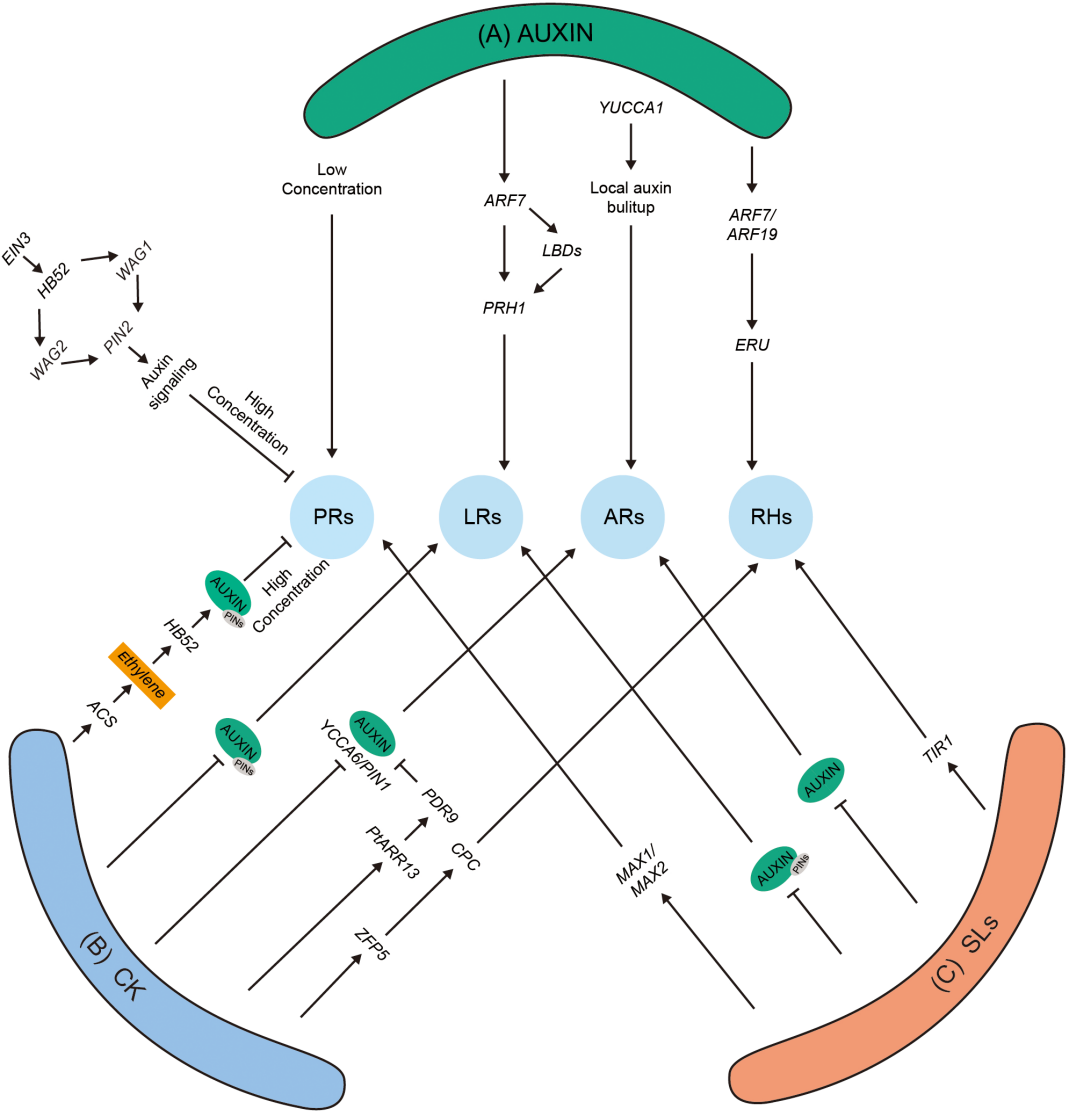
The role of auxin and its hormonal cross-talk with other phytohormones. **(A)** High auxin level negatively affects the PR length. *HB52* binds to the promotor of *WAG1*, *WAG2*, and *PIN2* and positively modulates their transcriptional levels. As a result, more auxin is transported to EZ. Auxin promotes LR development by inducing *RPH1* expression dependent on *ARF7* and *LBD*s. The overexpression of *YUCCA* or exogenously applied auxin promotes ARs in *Arabidopsis*. Auxins positively promote RHs by *ERU*-dependent (ERULUS is a core regulator for RHs growth) pathway. **(B)** CK positively regulates *ACS* gene, which produces more ethylene, and ethylene stabilizes *PIN2*, resulting in the modulation of more auxin in the roots, which further negatively regulates PRs. Application of CK negatively modulates LRs with disturbance of auxin levels via*PIN* expression. The application of CK regulates auxin levels in ARs by negatively modulating *YUCCA*, *ARR13*, and *PIN* genes. CK-induced RHs via CPC-mediated complex. **(C)** Exogenous application of SLs promotes PRs through *max*-dependent pathway. SL modulates LRs and ARs by altering auxin levels in roots in *Arabidopsis*. Under low Pi environments, SLs promote the expression of *TIR* gene, increasing auxin levels and promoting RHs. The local auxin accumulation in RSA promotes PR, LR, AR, and RH growth and development. *ACS*, *ACC synthase 2*; ARF, auxin response factor; ARs, adventitious roots; CK, cytokinins; EZ, elongation zone; *EIN3*, *ethylene-insensitive3*; PRs, primary roots; LRs, lateral roots; HB52, homeobox protein 52; *LBD*, lateral organ boundaries domain; PIN, pin formed; PDR, pleiotropic drug resistance transporter; WAG, wavy root growth; RPH1, leaf rust resistance gene; *TIR*, transport inhibitor response 1; RHs, root Hairs; *zfp*, *zinc finger protein 5*.

The three-dimensional structure of the plant root is known as the root system architecture (RSA), which consists of PRs, the numbers and patterns of LRs, adventitious roots (ARs), and RHs (**Figure 1A**). The RSA in different plant species exhibits high differences in the morphological traits, which help plants adapt to competitive environmental conditions. The cross-talk among phytohormones includes intricate and complex molecular mechanisms that regulate RA in plants (**Figure 2**). Furthermore, two different kinds of root systems exist in plants tap roots and fibrous roots, which are defined by their branching patterns.

In RSA, the PRs constitute the fundamental component that originates from the embryonic meristematic tissues close to the early globular embryo’s base and are the first to emerge. These PRs exhibit distinct developmental regions in RA (Svolacchia et al., 2020). At the root apical meristem, all the cells originate from stem cells or precursors within the stem cell niche (SCN), which is located in the tip. After that, daughter cells undergo differential rates of division and proportions, delineating a zone known as the division zone (DZ) (**Figure 1B**). Upon exiting the cell division cycle, these cells elongate and generate the zone of elongation (EZ) (Svolacchia et al., 2020). Subsequently, these cells differentiate, acquiring characteristics of specific tissues, based on their radical position, leading to the differentiation zone’s constitution. In RSA, the transition zone (TZ) assists as a developmental boundary between the division and differentiation of the cells. Furthermore, each of the developmental zones corresponds to a classified distribution of phytohormones, peptides, mRNA, and transcription factors. Notably, the functions of cytokinin and auxin are prominent in SCN and help in the facilitation of cell differentiation. The auxin gradient was noted higher in SCN and gradually decreased toward the TZ (Galinha et al., 2007).

LRs contribute to enhanced soil anchorage, promote efficient nutrient absorption and water uptake, and foster symbiotic relationships with microorganisms (Du and Scheres, 2018; Motte et al., 2019). The development of LRs mainly directed by the auxin activity, response maxima, and downstream signaling pathways govern the pattering of LRs in angiosperms. LR formations take place within the single-layered pericycle tissues located in the xylem poles and are known as xylem pole pericycle cells. Through continuous cell elongation and anticlinal division, xylem pole pericycle cells switch from the meristem to auxin-rich zones (Jansen et al., 2012). In addition, transcriptomic studies of LR initiations pointed out some common pathways in maize and *Arabidopsis*, which may suggest that conserved genes are involved in the LR initiation in angiosperms (Jansen et al., 2013).

AR roots are the roots that ascend from any other point than the radicle (embryonic roots) or the root axis of the plants; some can ascend from the stem tissues (Steffens and Rasmussen, 2016). These can manifest as crown roots, arising from the underground nodes, or the brace roots, which develop from the nodes that are above the ground as well as seminal roots and stem roots. Various root categories serve different structural and absorptive roles in plants. PRs and seminal roots are pivotal during the initial phases of plant growth, whereas brace roots become significant in the later stage of monocot RA and provide structure support to the plants (Steffens and Rasmussen, 2016).

RHs are elongated, tip-growing structures from the epidermis of roots, playing an important role in increasing the surface area of roots for enhancing nutrient and water absorption (Miguel et al., 2015). Additionally, they also promote root adhesion to the surrounding rhizosphere and facilitate soil, plant, and microbial interactions. Several studies have demonstrated the compromised fitness of plants with RH mutants or loss of function when they were subjected to harsh environmental conditions in soil (Schoenaers et al., 2018). Thus, the dynamic development of RH offers an opportunity for agricultural exploitation, enhancing nutrient and water acquisition in diverse soil environments. The initiation of RH development commences with positional signaling originating from the roots’ cortical cells. Overall, the above evidence suggests that environmental conditions and phytohormones in various developmental stages and tissues of RA significantly regulate root development.

## The intricate role of auxin and its interaction in root architecture

Previous studies have demonstrated a strong relationship between root development and auxin; therefore, it was named root-forming hormone (Zhu et al., 2019; Moreno-Risueno et al., 2010). Auxin showed a regulatory effect on almost every phase of root development and growth (**Figure 2A**). PR is stimulated in maize, *Arabidopsis*, tomato, and rice under low auxin concentrations (**Figure 2A**). In comparison, inhibited growth was observed at high concentrations through the *TRANSPORT INHIBITOR RESPONSE 1* (*TIR1*) receptor, mediating signaling by an unknown non-transcriptional mechanism (Fendrych et al., 2018). It has been noted that free IAA/Aux can positively regulate root development; in contrast, ubiquitinated Aux/IAA proteins can negatively regulate root development. Compared to other signals in root development, auxins are considered the most essential hormone that instructs root meristem and organogenesis (Fendrych et al., 2018). It was shown that auxin is produced through a tryptophan-dependent pathway in roots, in which tryptophan converts into indole acetic acid (IAA). *TRYPTOPHAN AMINOTRANSFERASE RELATED 2* (*TAR2*) protein and its homologous *WEAK ETHYLENE INSENSITIVE 8* (*WEI8*) are the two key enzymes that are involved in producing and maintaining the auxin homeostasis in roots. Mutants of *WEI8/TAR2* showed lower auxin concentrations and a loss of the meristem zone in plant roots (Brumos et al., 2018). Additionally, the roots deficient with auxin levels could not be recovered with shoot-derived auxins through a grafting experiment (Brumos et al., 2018).

In *Arabidopsis*, a gene *Nitrilase 1* (*NIT1*) coding for *nitrilase 1* enzyme modulates the auxin synthesis, and the overexpression of *NIT1* is responsible for shorter PR because of changes in IAA and IAN levels. A study on rice showed that *FPF1-like protein* (*OsFPFL4*) regulates the auxin levels and root growth. Overexpression of *OsFPFL4* resulted in shorter PRs because of the high endogenous accumulation of auxin and also altered the expression level of auxin’s biosynthesis and transporter-related genes (Guo et al., 2020b). Additionally, a gene *MADS-box Transcription Factor* (*OsMADS25*) activates the biosynthesis and transport of auxin in rice, modulating root development and growth (Zhang et al., 2018). Moreover, the mutant lines of (rice auxin influx carrier) *OsAUX3-1* and *OsAUX3-2* resulted in shorter PRs by changing the concentration of auxin in rice roots (Nan et al., 2014). Furthermore, in rice, the *OsWOX4* (WUSHEL-related homeobox protein) has been proven to be responsible for root elongation by transcriptional activation of *OsAux1* gene (Chen et al., 2020). The soybean gene *GmYUC2a* is considered an important regulator gene for root development and growth by regulating auxin biosynthesis (Wang et al., 2019). The auxin biosynthesis in roots can be regulated by other alternative mechanisms, such as exogenously applied chemicals and protein phosphorylation (Nikonorova et al., 2021). A study on exogenously applied cyclic guanosine 3′,5′-monophosphate (cGMP) provided evidence that PR growth in *Arabidopsis* was modulated due to IAA/AUX protein degradation (Nan et al., 2014). The phosphoproteome studies by Nikonorova et al. (2021) identified some new growth regulators/inhibitors, such as MAP kinases and some receptor-like kinases, by applying different concentrations of auxins and suggesting that cell wall modification, H+-ATPases, cell wall sensing receptors, and auxin concentration are responsible for the elongation of the cell root tips. It has been previously reported in many studies that auxin interacts with other phytohormones to govern the development of roots (**Figure 2**). The gene homeobox protein (*HB52*) has been shown to regulate the cross-talk between auxin and ethylene and modulate PR growth. *HB52* acts as the downstream of *ethylene-insensitive3* (*EIN3*) and also modulates the expression level of auxin transporters like *PIN2* and *WAVY ROOT GROWTH* (*WAG1* and *WAG2*) by binding with their promoters. The genes *WAG1* and *WAG2* phosphorylate *PIN2*, which gathers in the apical side of the root’s cells and results in more auxins being transported to the TZ, and because of the higher accumulation of auxins, the PRs were noted to be shorter (**Figure 2A**) (Mao et al., 2016). In addition to auxin’s transporters, the ethylene signaling gene *ETHYLENE RESPONSE FACTOR* (*ERF1*) was noted to regulate the auxin biosynthesis genes (*YUCCA*s) and *ANTHRANILATE SYNTHASE* alfa 1 (*ASA1*) during root development (Mao et al., 2016).

Few previous studies already discussed the interaction of cytokinin (CK) with auxin in modulating the cell architecture in the initial stages of root growth and development. In *Arabidopsis*, a newly identified *AKH3* transmembrane receptor belonging to the CK family, in conjunction with type B *ARABIDOPSIS RESPONSE REGULATOR* (*ARR1* and *2*), plays a vital role in the redistribution of auxins in the root’s meristem (Ioio et al., 2008a). They also provided evidence that changes in the auxin distribution are due to the modulation in the transcript level of *PIN* genes via the activation of auxin repressor genes like *SHORT HYPOCOTYL2* (*SHY2*) and *IAA3*, resulting in cell differentiation. Another study showed that CK modulates the transcription of *PIN* gene through the involvement of *CYTOKININS RESPONSE* FACTORS (*CRFI*), which degrades *SHY2*, resulting in the upregulation of *PIN* genes (Ioio et al., 2008b). Therefore, CKs are important for the homeostatic modulation of *SHY2* factors and the root architecture and development. The octuplet mutant of *arrs* (*3*, *4*, *5*, *6*, *7*, *8*, *9*, and *15*) showed smaller meristem size as compared to control plants and was highly sensitive to NPA inhibitor, which targets the auxin transporters (Zhang et al., 2011). Furthermore, when CK was applied externally to *arr* mutants, the reduced *PIN4-GFP* was noted in the root cap (Zhang et al., 2011).

Auxin-induced LR development has been widely studied in *Arabidopsis* and tomato by modulating their auxin signaling and biosynthesis genes; however, few studies claimed that a lower concentration of auxin (25 nM) reduced LR growth when applied externally (Banda et al., 2019). A canonical downstream target (*LBD16/ASYMMETRIC LEAVES2-LIKE18*; *ASL18*), that is, the conventional downstream target of (*SOLITARY ROOT (SLR)/IAA14-ARF19-ARF7*) auxin signaling module, modulates the growth and development of LRs, presumably through the transcriptional modulation via its associated *LBD/ASL*s, resulting in the asymmetric cell division in the initiations of LRs and primordium development (Goh et al., 2012). Some reports have documented that (*ATP-BINDING CASSETTE*) *ABCB* transporters can play a significant role in the emergence of LRs, and delayed emergence of LRs was observed in the mutants of *ABCB21* (Jenness et al., 2019). Similarly, these studies also suggest that *ABCB21* is also responsible for the modulation of auxin distribution and plays a role in LR formation. Based on earlier studies on *Arabidopsis*, it was noted that LR formation requires *ARF7*, and also *LATERAL ORGAN BOUNDARIES DOMAIN* (*LBD*), for normal development and growth. Recently, it was revealed that LR development in the *Arabidopsis* is modulated by *PRH1* through endogenous auxin concentration and the auxin signaling pathway. Furthermore, *PRH1* expression is induced by auxin, and reduced LRs were noted in *PRH1* mutant lines. Moreover, *PRH1* is shown as a direct transcriptional target of *LBD*s and *ARF7*, thus playing a positive role in cell wall loosening because it may able to regulate *EXPANSIN* (*EXP*) gene expression (**Figure 2A**) (Zhang et al., 2020).

Both LRs and ARs mostly use the same pathways; however, there are differences in some regulator processes. For example, *ARF7* and *ARF19* are responsible for regulating LRs and activating *LBD* genes, whereas for ARs, the TF *WOX11* overtakes the functions of *ARF*s. In procambium and its adjacent cells, *WOX11* responds to the wound-induced auxins and activates the downstream *LBD 16* and *29*, together with its homologous protein *WOX12*, regulating the fate transition from procambium to founder cells of the roots (Liu et al., 2014). Similarly, few researchers suggested that *ARF17* and *19* are responsible for the regulation of ARs in hypocotyls, which describes the exact molecular mechanism for AR and LR development (Wilmoth et al., 2005). Moreover, the overexpression line *35S:YUCCA1* resulted in higher numbers of ARs as compared to WT, which was consistent with 100 nM exogenously applied IAA, which can increase ARs in *Arabidopsis* (**Figure 2A**). Few studies have clearly defined the role of auxin transporters during AR development (**Table 1**). The AT-binding cassette *ABCB19* and *IBA* have been linked to inducing AR formation. It has been suggested by a recent study that AR development is mediated by *ARF8*, *ARF17*, and *ARF6*, which further modulates the transcriptional levels of *GH3.3*, *3.5*, and *3.6*; it has been documented that *ARF6* and *8* are the two positive modulators, while *ARF17* acts as a negative regulator (Gutierrez et al., 2012). In addition, these three genes control JA homeostasis and lead to an increase in JA-lle, which activates the JA.lle/*COL1* signaling pathway and negatively modulates ARs in *Arabidopsis*. In conclusion, auxin modulates AR initiation by the activation of *ARF8* and *ARF7*, which results in AR ambitions through the *COL1* signaling pathway (Gutierrez et al., 2012).

**Table 1:** List of recently published genes involved in alteration of root development.

Gene name	Engineered species	Type	RSA traits	Reference
*CaCKX6*	*Cicer arietinum*	Overexpression	Increase LR	(Khandal et al., 2020)
*MsMiR156/MsSPL13*	*Medicago sativa*	Overexpression/RNAi	Increase ARs and biomass	(Arshad et al., 2017)
*PsNTP9*	*Glycine max*	Gain of function	Increase PRs, LRs, and RHs	(Veerappa et al., 2019)
*Specific GA2ox6*	*Oryza sativa*	Gain of function	Increase tillers and root system	(Lo et al., 2017)
*SIDWARF*	*Solanum lycopersicum*	Overexpression	Increase PRs length and LRs numbers	(Li et al., 2016)
*TabZIP60*	*Triticum aestivum*	RNAi	Increase LRs	(Veerappa et al., 2019)
*TaSNAC8-6A*	*T. aestivum*	Overexpression	Increase LRs	(Mao H. et al., 2020)
*ZmPINa*	*Zea mays*	Overexpression	Increase root growth	(Ogura et al., 2019)
*OsmiR164b-* *Resistant OsNAC2*	*O. sativa*	Overexpression	Increase roots	(Jiang et al., 2018)
*AtTPP1*	*Arabidopsis thaliana*	T-DNA inserted/Loss of function	Regulate PRs and LRs	(Lin et al., 2023)
*OsDOF15*	*O. sativa*	Overexpression	Increase PRs	(Qin et al., 2019b)
*OsNAC2*	*O. sativa*	Overexpression	Regulate root length	(Mao C. et al., 2020)
*AtLRP1*	*A. thaliana*	Overexpression	Increase LRs primordium	(Singh et al., 2020)
*OsRLR4*	*O. sativa*	Overexpression	Regulates PRs	(Sun et al., 2022)
*ARF4*	*T. aestivum*	Ectopic	Root growth	(Wang et al., 2019)
*LBD16*	*T. aestivum*	Diploid parents	LR development	(Wang et al., 2018)
*AB14*	*A. thaliana*	Overexpression	Regulate PRs	(Luo et al., 2023)
*PLATZ17*	*G. max*	RANi	Hairy roots	(Zhao et al., 2022)
*GTL1*	*A. thaliana*	T-DNA inserted, mutant	Regulate RHs	(Shibata et al., 2022)
*SIGH3.15*	*S. lycopersicum*	Overexpression	Regulates LRs	(Ai et al., 2023)
*LCO-responsive homolog of NIN2*	*Populus* sp.	RNAi	Regulates LRs	(Irving et al., 2022)
*CLE3*	*A. thaliana*	Overexpression	Regulates LRs	(Nakagami et al., 2023)
*DA3*	*A. thaliana*	RNAi	Regulates LRs	(Peng et al., 2023)

GA2ox6, GA2-oxidase 6; PsNT9, Pisum sativum nucleoside triphosphate diphosphohydrolase 9; AtTPP1, trehalose-6-phosphate phosphatase; DOF, DNA-binding with one finger; LRP1, lateral root primordium1; RLR4, root length regulator; ARF4, auxin response factor 4; LBD16, lateral organ boundaries domain 16; AB14 ABA insensitive4 (AB14)-cyclin-dependent kinase B2; PLATZ, plant AT-rich sequence and zinc binding TF; GTL1, trihelix transcription factors GT2-Like1; GH3, Gretchen Hagen 3; NIN, Nodule Inception TFs; CLE, CLAVATA3 (CLV3)/EMBRYO SURROUNDING REGION-RELATED; DA3, ubiquitin-specific proteases 14.

Another essential role of auxin in the RSA is the development and growth of RHs (Hossain et al., 2015). A new study on trichoblasts explained that the expression level of (*ROOT HAIR DEFECTIVE 6 LIKE 4*) *RSL4* is controlled by *ARF5*, *ARF7*, *ARF8*, and *ARF19* by binding with its promoters. In addition, it was also noted that auxins control the modulation of cell wall receptor protein ERULUS (the member of *Catharanthus roseus receptor Like-Kinase-1-Like*) in an *ARF19*- and *ARF7*-dependent manner (**Figure 2A**) (Schoemaers et al., 2018). The overexpression of *ARF1–4* and *ARF16* negatively modulated the growth of RHs and suggested the different roles of *ARF*s in RHs. Furthermore, ethylene and auxin were demonstrated to regulate RH growth and development synergistically (Bruex et al., 2012). Taken together, auxin biosynthesis, transport, and concentration are important RSA.

## Cytokinins significantly regulate root architecture

CK is another phytohormone that plays a vital role in forming PRs. Exogenous application or high endogenous concentration of CKs inhibits proper root development by negatively affecting the cell division in the apical meristem of roots (Tiwari and Bhatia, 2020). Similarly, CK signaling mutants of type A *ARR* and type B *ARR* showed a strong impact on PR length in some monocots and dicots (Gao et al., 2014; Márquez et al., 2019). Furthermore, single or double CK-deficient mutants showed an increased root phenotype (Werner et al., 2003; Higuchi et al., 2004).

Cytokinin–auxin interaction regulates plant growth in numerous plant species, i.e., rice, tomato, and maize (Kurepa et al., 2018). CK performs a crucial role in the modulation of cell differentiation by enhancing the positive effects of auxins on cell division (Ioio et al., 2007; Ioio et al., 2008a. Through the *ARR1*/*AKH3* pathway, CK activates the transcription of auxin/IAA and *SHY2*/*IAA3* genes within vascular tissues of the TZ (Ioio et al., 2008b). *SHY2* negatively influences *PIN* expression and changes the auxin transport to roots (Ioio et al., 2008a). In addition, the overexpression of *SHY2-2* resulted in impaired root growth upon exogenous CK application, and loss of function resulted in higher root length, which is closely similar to *arr1* mutants. Furthermore, the *PIN* expression was downregulated in *shy2-2* mutant lines, providing a relationship among *SHY2*, *PIN*s, and root development (Ioio et al., 2008a). Moreover, CK induces ethylene biosynthesis by stabilizing the *ACS* gene; ethylene through *HB52*, *WAG1*, and *WAG2* stabilizes *PIN2* gene and inhibits PR growth due to elevated auxin levels (**Figure 2B**) (Qin et al., 2019a). In summary, these findings suggested that the plants require minimum CK and auxin levels for normal development of PRs.

Concurrently, CK negatively regulates LR formation in plants. Increased levels of endogenous level or exogenously applied CK inhibit the LRs in rice and *Arabidopsis* (Debi et al., 2005; Li et al., 2006). In comparison to the mutant plants of the *AKH* and type B *ARR*, the gain of function displayed enhanced LR formation (Werner and Schmülling, 2009; Del Bianco et al., 2013). Similarly in *Arabidopsis*, some type A and type B *ARR* mutants displayed reduced LR numbers as compared to the control (To et al., 2004). It was noted that the reduction of LRs through CK was because of modulation in auxin transport and the disturbance of *PIN1* localization (Mason et al., 2005). Exogenously applied CK rapidly regulated *PIN1* gene expression, further clarifying CK’s role in modulating auxin transport (**Figure 2B**) (Laplaze et al., 2007). In addition, a recent report identified a new *TRANSPORTER OF IBA* (*TOB1*) gene, which is highly expressed in lateral root caps and showed repose to CK. *TOB1* is responsible for transporting IBA in the cytoplasm of the cell, which converts into active form IAA, and the mutant of tob1 increased the production of LR number (Michniewicz et al., 2019).

Numerous studies have explored the interaction between CK and auxin in modulating the development and growth of ARs in many species (**Table 1**). Additionally, in *Arabidopsis*, CK showed negative influences on *PIN* (like Aux1), *LAX3*, and *YUCCA6* genes, resulting in the alteration of auxin flow and repression of ARs (**Figure 2B**) (Li, 2021). In *Populus tremula*, *PtRR13* modulates CK signaling and results in AR repression. *PtRR13* modulates the expression of *PLEIOTROPIC DRUG RESISTANCE TRANSPORTER* (*PDR9*), which is responsible for the auxin’s efflux pump, which shows further impact on the vascular tissue formation in AR rooting, thus confirming the role of CK and auxin cross-talk (**Figure 2B**) (Ramirez-Carvajal et al., 2009). Furthermore, *PtRR13* also inhibits AR development and growth by negatively modulating two ethylene-responsive *TINY-like* transcription factors, bridging the gap between CK and ethylene (Ramirez-Carvajal et al., 2009). In addition, AR rooting culture for *Medicago truncatula* CK downregulated the expression of *PLETHORA* (*PLT2* and *PLT1*) while upregulating the expression level of (*AINTEGUMENTA-like*) *AIL1*, *WOX4*, and *AINTEGUMENTA* (*ANT)* and the *SHOOT MERISTEMLESS* (*STM*) transcriptional factors. In poplar, *WOX* genes like *Pewor11b* or *Pewox11a* enhanced AR formation, with *wox11* acting as a mediator between CK and auxin by downregulating type B CK response regulators (Kitomi et al., 2011; Xu et al., 2015; Zhao et al., 2009). These findings elucidate the interaction of CK and auxin pathways in AR development.

Similarly, a recent article suggested the positive role of CK in root hair elongation (Takatsuka et al., 2023). It noted that RH elongation involves type A *ARR*s, *ARR12* and *ARR1*; *ARR1* can directly induce the transcriptional level of *RSL4*, which plays an important role in CK-dependent RH development (Takatsuka et al., 2023). Exogenously applied CK promoted RH elongation, whereas the overexpression of cytokinin oxidase 2 (*CKX2* code for CK degrading) displayed shorter RH formation (Zhang et al., 2016). In *Arabidopsis*, some TFs regulate RH growth and development, and mutants of *zinc finger 5* (*zfp5*) displayed fewer root hairs than WT. The modulation in the expression level of *ZFP5* was increased by the application of 6-benzylaminopurine, which is known as RH inducer. *ZFP5* has the ability to induce the transcriptional level of MYB proteins, CAPRICE (CPC), which binds to their promotors that are involved in the RH patterning. Furthermore, this complex inhibits the negative modulators of root hairs like GLARBAI (GL2), as shown in **Figure 2B** (Wada et al., 2002).

## Strigolactones decide the fate of RA

The roots in plants are considered the central part of strigolactone (SL) biosynthesis (Dun et al., 2009). Numerous studies suggested that SL interferes with the auxin’s transporters to regulate root growth and development (Xie et al., 2010, Rasmussen et al., 2012; Xie et al., 2015). In different plant species, several mutants of SL biosynthesis (*max4* and *max3*) and signaling (*D14* and *D3*) have been identified (Claassens and Hills, 2018). SL promotes PRs and AR growth, but a negative effect has been noted in LR growth (**Figure 2C**) (Kumar et al., 2015; Sun et al., 2016). At lower concentrations, GR-24 has been shown as an LR growth and development enhancer, while higher concentrations (2.5 μM) reduced LR growth in a few species (Jain et al., 2007; Shinohara et al., 2013).

In *Arabidopsis*, the PR length was reduced because of smaller numbers of cells in the meristem zone of the roots in the mutant lines as compared to the wild type (Ruyter-Spira et al., 2011). Under specific conditions, the biosynthesis mutant of SL, *max1* (*cytochrome P450*), and *max4* (*carotenoid cleavage dioxygenase*), *max3*, and *d14* were observed with shorter PR lengths as compared to WT. Exogenous application of GR-24 recovered PRs in the *max4* mutant line, while signaling mutants showed no significant change in roots (Ruyter-Spira et al., 2011). Similarly, PR recovery was noted in SL-deficient mutants in tomato, rice, and *M. truncatula* after the application of GR-24, while no change or non-significant changes were noted in WT or signaling mutants (Koltai et al., 2010; Sun et al., 2014; De Cuyper et al., 2015).

Exogenously applied GR-24 treatment appears to inhibit LRs in numerous plant species (Arite et al., 2012; Marzec et al., 2016; and De Cuyper et al., 2015). A signaling mutant of SL (*hvd14.d*) in *Hordeum vulgare* L. was noted with higher lateral root density, while the change in the root length was non-significant as compared to the wild type (Marzec et al., 2016). It was observed in *Lotus japonicus* L., in which the SL-biosynthesis mutant line *carotenoid cleavage dioxygenase* 7 (*LjCCD7*) showed more LRs than WT (Liu et al., 2013). SL may affect LRs by changing the concentration of auxin in roots. The application of GR-24 affects the *PIN* auxin efflux in roots, which results in reduced PIN-GFP intensity in LRs (**Figure 2C**) (Koltai et al., 2010; Ruyter-Spira et al., 2011).

In addition, both SL-deficient and signaling mutants in pea and *Arabidopsis* resulted in more ARs as compared to WT, which shows that SL interferes with AR formation (**Figure 2C**) (Srivastava et al., 2018). To confirm the interaction between auxin and SL, Rasmussen et al. examined the SL response to an over-producing line of auxin *35:YUCCA1* and demonstrated that the over-producing line increased AR formation as compared to WT, which is in line with the property of auxin, which promotes ARs. In contrast, the *35:YUCCA1* line treated with exogenous application of SL reduced AR formation by up to 50%. In the case of the *max3* mutant treated with IAA, IBA produced more ARs as compared to the control. Similarly, ARs were decreased in pea and *Arabidopsis* mutants, and further investigation of *CYCLIN B1* confirmed that SL decreased the number of ARs by interfering in the cell division of founder’s cells (Rasmussen et al., 2012; Brewer et al., 2013). Interestingly, in rice, SL was shown to positively regulate ARs (Arite et al., 2012; Sun et al., 2014). Furthermore, SL mutants with biosynthesis and signaling in rice showed fewer ARs in the seedling and tillering stage (maturation stage). Treatment with exogenously applied GR-24 recovers the ARs in the (*d10*) biosynthesis mutant while remaining unchanged in the signaling mutant (Sun et al., 2015). In addition, maize (*Zea mays*) SL-deficient mutant *zmccd8* resulted in shorter nodal ARs as compared to the control plants, which shows the regulator role of SL in AR development (Guan et al., 2012). Altogether, these consequences suggest that SLs can negatively regulate the AR development in eudicot plants like *Arabidopsis*. In contrast, increased number and elongation of ARs were noted in grasses like maize and rice plants. Auxin signaling is important for normal AR development, and SL regulates the auxin levels in the pericycle and modulates AR initiation (Rasmussen et al., 2012).

Exogenously applied GR-24 was noted to increase RHs in tomatoes and *Arabidopsis* (Koltai et al., 2011; Kapulnik et al., 2011). It was demonstrated that SLs and ethylene modulate root hair elongation. Furthermore, the treatment of SLs increased the expression of *ACC synthase 2* (*ACS2*) gene and activated the acs enzyme, suggesting a shared regulatory pathway for RH development. Additionally, SLs were noted to regulate RH elongations through an auxin-dependent pathway (Kapulnik et al., 2011; Kohlen et al., 2012). A study has shown that phosphate deficiency promotes SL accumulation and induces the regulation of auxin receptor gene *TIR1* in the WT plants but not in the *max4*-deficient mutant, signifying that under specific conditions, both SL and auxins affect the root hair density (**Figure 2C**). In summary, SLs are proposed to modulate auxin transport and regulate RSA.

## Conclusion and future perspectives

This review emphasizes the complexity of plant phytohormones in RSA and highlights hormonal effects depending on specific combinations or individual actions. This study explores cross-talk between various hormones like auxin–SL and auxin–CK during different parts of RSA, indicating that different hormones coincide in regulating different processes in plants. Indeed, ample evidence demonstrates that the response of phytohormone changes during various stages of organ development, potentially due to diverse cellular and tissue environments. The configuration of root morphology is influenced by a highly complex network of phytohormones, highlighting the importance of comprehending key transcriptional and post-transcriptional pathways, along with their associated genes. Mapping these genes could offer insights into hormonal regulation and developmental outcomes. More systemic investigations and computational models are crucially needed to understand the hormonal cross-talk for root development under realistic field conditions. These investigations will not only help to consolidate the knowledge about how roots perceive internal and external signals and translate them into cellular responses but will also enable breeders to create predictive models to pinpoint essential regulators and integrators for governing RSA during various environmental conditions.

## Author contributions

MJ: Conceptualization, Investigation, Methodology, Project administration, Software, Writing – original draft, Writing – review & editing, Validation. MS: Conceptualization, Data curation, Investigation, Methodology, Writing – original draft, Writing – review & editing, Project administration, Resources, Visualization. WJ: Conceptualization, Data curation, Investigation, Methodology, Software, Writing – review & editing. WZ: Conceptualization, Formal Analysis, Investigation, Resources, Visualization, Writing – original draft. SZ: Data curation, Formal analysis, Resources, Software, Visualization, Writing – original draft. YL: Conceptualization, Data curation, Funding acquisition, Investigation, Project administration, Resources, Validation, Writing – original draft. YZ: Conceptualization, Data curation, Formal analysis, Writing – original draft. JL: Conceptualization, Data curation, Formal analysis, Investigation, Methodology, Project administration, Software, Writing – review & editing. HL: Conceptualization, Data curation, Software, Supervision, Writing – original draft. RM: Conceptualization, Data curation, Software, Validation, Writing – original draft. QY: Formal analysis, Project administration, Software, Supervision, Writing – original draft. MA: Methodology, Project administration, Visualization, Writing – original draft, Writing – review & editing. GW: Conceptualization, Data curation, Funding acquisition, Project administration, Resources, Supervision, Validation, Visualization, Writing – original draft.
